# Distinct Origins and Transmission Pathways of *bla*_KPC_ *Enterobacterales* across Three U.S. States

**DOI:** 10.1128/jcm.00259-23

**Published:** 2023-07-13

**Authors:** Zena Lapp, Rany Octaria, Sean M. O’Malley, Tu Ngoc Nguyen, Hannah Wolford, Ryan Crawford, Christina Moore, Paula Snippes Vagnone, Diane Noel, Nadezhda Duffy, Ali Pirani, Linda S. Thomas, Brittany Pattee, Claire Pearson, Sandra N. Bulens, Sophie Hoffman, Marion Kainer, Melissa Anacker, James Meek, Isaac See, Kyle J. Gontjes, Allison Chan, Ruth Lynfield, Meghan Maloney, Mary K. Hayden, Evan Snitkin, Rachel B. Slayton

**Affiliations:** a Department of Computational Medicine and Bioinformatics, University of Michigan, Ann Arbor, Michigan, USA; b Department of Medicine, Division of Epidemiology, Vanderbilt University, Nashville, Tennessee, USA; c Tennessee Department of Health, Nashville, Tennessee, USA; d Minnesota Department of Health, Saint Paul, Minnesota, USA; e Connecticut Department of Public Health, Hartford, Connecticut, USA; f Centers for Disease Control and Prevention, Atlanta, Georgia, USA; g Department of Microbiology and Immunology, University of Michigan, Ann Arbor, Michigan, USA; h Connecticut Emerging Infections Program, Yale School of Public Health, New Haven, Connecticut, USA; i Department of Medicine, Division of Infectious Diseases, Rush University Medical Center, Chicago, Illinois, USA; j Department of Pathology, Rush University Medical Center, Chicago, Illinois, USA; Maine Medical Center Department of Medicine

**Keywords:** transmission, carbapenem-resistant *Enterobacterales* (CRE), importation, patient transfer

## Abstract

Carbapenem-resistant *Enterobacterales* (CRE) are among the most concerning antibiotic resistance threats due to high rates of multidrug resistance, transmissibility in health care settings, and high mortality rates. We evaluated the potential for regional genomic surveillance to track the spread of *bla*_KPC_-carrying CRE (KPC-CRE) by using isolate collections from health care facilities in three U.S. states. Clinical isolates were collected from Connecticut (2017 to 2018), Minnesota (2012 to 2018), and Tennessee (2016 to 2017) through the U.S. Centers for Disease Control and Prevention’s Multi-site Gram-negative Surveillance Initiative (MuGSI) and additional surveillance. KPC-CRE isolates were whole-genome sequenced, yielding 255 isolates from 214 patients across 96 facilities. Case report data on patient comorbidities, facility exposures, and interfacility patient transfer were extracted. We observed that in Connecticut, most KPC-CRE isolates showed evidence of importation from outside the state, with limited local transmission. In Minnesota, cases were mainly from sporadic importation and transmission of *bla*_KPC_-carrying Klebsiella pneumoniae ST258, and clonal expansion of *bla*_KPC_-carrying Enterobacter hormaechei ST171, primarily at a single focal facility and its satellite facilities. In Tennessee, we observed transmission of diverse strains of *bla*_KPC_-carrying Enterobacter and *Klesbiella*, with evidence that most derived from the local acquisition of *bla*_KPC_ plasmids circulating in an interconnected regional health care network. Thus, the underlying processes driving KPC-CRE burden can differ substantially across regions and can be discerned through regional genomic surveillance. This study provides proof of concept that integrating genomic data with information on interfacility patient transfers can provide insights into locations and drivers of regional KPC-CRE burden that can enable targeted interventions.

## INTRODUCTION

Carbapenem-resistant *Enterobacterales* (CRE) accounted for an estimated 13,100 infections among hospitalized patients in the United States in 2017 (1) and likely colonized upwards of 100,000 additional patients (2). Infections with CRE are an urgent public health threat as they can be difficult to treat due to resistance to carbapenems and other last-resort antibiotics (3, 4). Of concern are CRE containing a carbapenemase gene found on a mobile genetic element, which can disseminate within and between different species via horizontal gene transfer (HGT), followed by subsequent clonal dissemination (5). Implementing interventions that effectively reduce CRE infections and transmission requires not only monitoring regional CRE infections, but also understanding where they were acquired. In particular, because of the capacity for CRE to colonize patients for months and even years (6), the facility where a patient developed an infection may not be where they acquired it. As active surveillance for CRE colonization at a regional level is logistically infeasible, strategies are needed to leverage clinical isolate collections to discern the origin of patient’s CRE infections in near real time.

Understanding whether and how a hospitalized patient’s CRE strain is related to previous cases is critical for effective and efficient surveillance and intervention strategies. The CRE burden in some regions appears to be driven by the evolution or importation of lineages with epidemic potential, such as *bla*_KPC_-carrying Klebsiella pneumoniae (KPC-Kp) ST258 (e.g., in California) (5) and Enterobacter hormaechei (KPC-Eh) sequence type 171 (ST17)1 (e.g., in Minnesota) (7) in the United States. Following initial regional emergence, these strains may be transmitted within health care facilities and spread between facilities via the transfer of colonized patients (5, 8, 9). In regions with sustained transmission of epidemic clones, effective regional control requires the identification of locations where transmission is occurring and impacting the burden in the region and monitoring the movement of CRE carriers between health care facilities (10, 11). In contrast, others have observed a role for HGT of mobile carbapenemase elements in driving CRE spread within individual health care facilities (12–14). If HGT is a significant contributor to the overall regional CRE burden, effective prevention and control require the identification of reservoirs of these resistance elements and an understanding of the propensity of different strain-mobile element combinations to spread between patients or act as HGT donors.

Here, we evaluate the potential for passive regional genomic surveillance to inform the pathways leading to CRE cases across regional health care networks. To do so, we used samples collected from three U.S. states involved in the U.S. Centers for Disease Control and Prevention’s (CDC) Multi-site Gram-negative Surveillance Initiative (MuGSI). In particular, we focus on the most commonly observed CRE in these regions during the study period, KPC-Kp and KPC-Eh. This builds off the MuGSI aim to quantify the burden of certain resistant Gram-negative bacteria in the United States. by supplementing preexisting data with whole-genome sequencing (WGS) of CRE isolates collected through MuGSI and supplemental statewide surveillance. By integrating genomic data from densely sampled CRE cases and health care exposure data and comparing local strains with global isolates, we show how these data can provide insight into local strain origins and regional transmission pathways.

## MATERIALS AND METHODS

This retrospective study was a collaboration between the U.S. Centers for Disease Control and Prevention (CDC) Emerging Infections Program (EIP) and CDC Prevention Epicenters. The EIP is a collaboration between 10 state health departments and their partnering academic institutions, the CDC, and other federal agencies (15). The Healthcare-Associated Infections Community Interface (HAIC) is one of the core components of the EIP and conducts active population- and laboratory-based surveillance for CRE (16). Three EIP sites participated in this study: Connecticut, Minnesota, and Tennessee.

### Catchment areas.

The Multi-site Gram-negative Surveillance Initiative (MuGSI) (17) CRE surveillance catchment areas for each state participating in this study are (i) the entire state of Connecticut, (ii) Hennepin and Ramsey counties, encompassing the metropolitan Minneapolis-Saint Paul area, of Minnesota, (iii) and Tennessee Emergency Medical Services (EMS) region 5, an 8-county region encompassing the metropolitan Nashville area of Tennessee.

While only selected counties in Minnesota and Tennessee participated in the HAIC MuGSI (17), each of the participating states conducted passive statewide public health surveillance for CRE. This yielded statewide coverage of all health care facilities in Connecticut and Minnesota, and coverage of all health care facilities in Emergency Medical Services (EMS) regions 2 to 7 in Tennessee (76.6% of the population). We excluded counties in the Memphis-Delta and northeast Tennessee regions due to significant health care utilization across state lines. Table S1 in the supplemental material provides information on the number of each type of health care facility in each state and the number of each type for which we have at least one sequenced isolate.

### CRE surveillance.

All carbapenem-resistant isolates were submitted by clinical laboratories to each state’s respective State Public Health Laboratory (SPHL).

CRE are defined as any organism in the *Enterobacterales* order isolated from any clinical specimen and resistant to doripenem, meropenem, or imipenem at MICs of ≥4 μg/mL, resistant to ertapenem at a MIC of ≥2 μg/mL, or demonstrates production of a carbapenemase (17). Specifically, isolates from residents of the surveillance area identified at the clinical laboratory as CRE from Escherichia coli, Enterobacter cloacae complex species (i.e., E. cloacae, *E. asburiae*, *E. bugandensis*, *E. hormaechei*, *E. kobei*, *E. ludwigii*, and *E. nimipressuralis*), and Klebsiella species (i.e., K. aerogenes, K. oxytoca, and K. pneumoniae) were included. All CRE isolates identified through the public health surveillance systems were characterized at the respective SPHL, which tested for carbapenemase genes (e.g., *bla*_KPC,_ *bla*_NDM_, *bla*_OXA-48_, *bla*_VIM_, and *bla*_IMP_) by PCR. (See the supplemental Methods section for state-specific details).

CRE considered for WGS were collected in Connecticut from 2017 to 2018, in Minnesota from 2012 to 2018, and in Tennessee from 2016 to 2017 (see Fig. S1 in the supplemental material). Multiple isolates from the same patient were obtained if the new isolate was from at least 30 days after a previous case isolate was identified. Unless otherwise noted, only isolates from 2016 to 2018 were analyzed to enable more meaningful comparison of analyses across states. While these data may not be representative of the present epidemiological situation, they still allow us to determine what types of data and analyses are useful to inform current and future public health surveillance and intervention efforts.

### Epidemiologic data.

Epidemiologic data were obtained through medical record review and in Tennessee supplemented by additional data sources. Isolate metadata include state, treatment facility ID, patient ID, age, sex, culture source, and hospitalizations in the previous year. MuGSI surveillance data also included information on infection type and underlying conditions, which was only obtained for selected non-MuGSI isolates. The only time we used this additional data was to investigate shared underlying conditions; this analysis was limited to samples for which we had data on these conditions. All other analyses included all isolates. The supplemental Methods section contains more information about how each state extracted the epidemiological information.

### Generation of aggregate patient transfer networks.

Aggregate patient transfer networks (i.e., patient flow across health care facilities) (5) for each state for 2017 were derived from Centers for Medicare & Medicaid Services (CMS) fee-for-service beneficiary claims data linked to the CMS minimum data set by Medicare beneficiary ID. The number of transfers between two facilities includes transfers directly from one facility to another and transfers with an intervening stay in the community of less than 365 days. The supplemental Methods section describes in detail how the patient transfer networks were generated by the CDC.

### Isolate and genomic data processing.

Over 90% of clinical CRE isolates considered for WGS were KPC-Kp or KPC-Eh; we therefore performed Illumina WGS on only this subset of isolates (see the supplemental material). For this set of isolates, we identified species and sequence types (STs) (18, 19), called single nucleotide variants (SNVs), using species-specific reference genomes (K. pneumoniae, KPNIH1, GenBank accession no. CP008827.1, 5,394,056 bp; *E. hormaechei*, MNCRE9, GenBank accession no. JZDE00000000.1, 4,911,317 bp) (20–26), built reference-based phylogenetic trees (25–29), and generated and annotated assemblies (20, 21, 30, 31). These reference-based data were used for all analyses unless otherwise noted. Furthermore, to prevent overcounting of intrafacility transmission, isolates were limited to the first isolate per patient for each ST-facility-patient combination. The supplemental Methods section contains details about how each state performed DNA extraction and sequencing and how the sequencing reads were processed.

### Identification of KPC-associated plasmids.

We used MOB-suite v.3.1.0 to reconstruct plasmid content from our genome assemblies (32). MOB-recon was used to reconstruct plasmid content, while MOB-typer performed plasmid typing and MOB-cluster assignment. Next, we identified KPC content using blastn v.2.13.0 and a curated BLAST database of all KPC alleles found in the Comprehensive Antibiotic Resistance Database (CARD) v.3.2.6 (33, 34). By using MOB-recon contig reports, KPC-containing contigs were mapped to their respective location (chromosomal or plasmid). Plasmid clusters where at least one isolate's KPC-containing contig was assigned to that plasmid call were classified as KPC-associated plasmid clusters. The proportion of KPC-associated plasmid cluster sharing across two species/STs was calculated by dividing the number of isolate pairs across the two categories that shared a KPC-associated plasmid cluster by the total number of isolate pairs.

### Identification of putative geographic importation events.

A total of 5,346 public KPC-positive and KPC-negative K. pneumoniae and *E. hormaechei* isolates were downloaded from the PATRIC database on 23 April 2021 (19, 35) and used to contextualize study isolates. In particular, we aimed to use geographic context provided by PATRIC isolates to group study isolates into clusters that could be traced back to common local emergence events based on phylogenetic clustering of isolates from the same state. Next, we used knowledge of *bla*_KPC_ presence in study and public genomes to assess whether a cluster likely originated via importation of a *bla*_KPC_-carrying strain from outside the state, versus local acquisition of *bla*_KPC_ via plasmid transfer.

For all public and study isolates, ST-specific core genome phylogenies were generated using cognac v.1.0 (25, 29, 36). The *bla*_KPC_ gene was identified for each isolate using the CD-HIT v.4.7 (37) data generated by cognac and gene annotations provided by RAST v.1.035 (35). For each core genome tree, maximum likelihood ancestral reconstruction of each study site location (Minnesota, Tennessee, and Connecticut) and the presence of *bla*_KPC_ was performed individually in R v.4.0.2 (38) using the ace() function in ape v5.5 (39). Nodes with an ancestral reconstruction confidence of <0.875 were discarded. All transition events (for each geographic location and *bla*_KPC_) were then mapped onto the edges of the phylogeny. Each KPC-positive study isolate tip was traversed toward the root of the tree until the first transition event was identified. If the first transition event was a geographic transition event with no *bla*_KPC_ transition event on the same edge, isolates were considered to originate from importation of a KPC-positive strain. If the first transition event was for *bla*_KPC_, this supports local acquisition of *bla*_KPC_ via HGT; therefore, these isolates were presumed to not originate from importation of a KPC-positive strain. If a geographic and *bla*_KPC_ transition occurred on the same edge, or if transition edges were of low confidence, we considered there to be no evidence of importation of a KPC-positive strain. Study isolates traced back to the same edge transition event and with the same KPC-associated plasmid cluster, regardless of the type or confidence of the transition, were considered part of a local cluster traced back to a common local emergence event. We consider the number of transition events for a given geographic location to be the number of identified clusters.

### Computing pairwise distances.

Pairwise single nucleotide variant (SNV) distances and patristic distances for study isolates from each species-specific reference-based sequence alignment were calculated in R v.4.0.2 (38) using the dist.dna() function in ape v5.5 (39) with pairwise.deletion = TRUE. These pairwise SNV distances were classified as intra- and interfacility pairs using the get_pair_types() function in regentrans v.0.1 (40). We expect there to be an enrichment in closely related intrafacility pairs under the assumption that intrafacility transmission is more common than interfacility transmission. Therefore, we plotted the proportion of intrafacility isolate pairs across pairwise SNV distances to select thresholds by identifying SNV distances where there is a decrease in the fraction of intrafacility isolate pairs (Fig. S3A) (40). Rather than choosing a specific pairwise SNV distance threshold, throughout the results we highlight various thresholds (≤5, 10, and 15 SNVs) as a sensitivity analysis to test how robust our observations were to different definitions of relatedness/recent transmission. For each threshold, we considered anything below that threshold to be related by recent transmission (potentially through an unsampled intermediate). A threshold of 5 was chosen for ST171 where needed based on a decrease in the fraction of intrafacility pairs after 5 SNVs.

### Integrated genomic and epidemiologic analyses. (i) Relatedness of intra- versus interfacility isolates.

For each isolate with at least one intrafacility isolate pair and at least one interfacility isolate pair, we identified the minimum pairwise SNV distance of all intrafacility pairs and the pairwise minimum pairwise SNV distance of all interfacility isolate pairs. We then compared the pairwise SNV distances of intra- versus interfacility isolate pairs using a paired Wilcox test.

### (ii) Intrafacility isolate pair shared comorbidity analysis.

For each intrafacility isolate pair, we determined whether they had at least one shared comorbidity and used a Wilcox test to compare pairwise SNV distances of patients who shared comorbidities and those who did not. This was done to test whether patients with shared comorbidities were a part of transmission subnetworks, possibly due to proximate inpatient locations where they may jointly be provided care.

### (iii) Interfacility isolate pair shared prior health care exposure analysis.

For each interfacility patient pair, we determined whether they had a shared health care exposure in the past year (data collection method described in the supplemental Methods section). Then we used a Wilcox test compared the pairwise SNV distances of patients who shared a health care exposure and those who did not.

### Data analysis and visualization.

R v.4.1.1 was used for all data analysis and visualization using the following packages: tidyverse v.1.3.1 (41), ggtree v.2.5.2 (42, 43), igraph v.1.2.6 (44), regentrans v.0.1 (40), tidygraph v.1.2.0 (45), ggraph v.2.0.5 (46), exact2x2 v.1.6.5 (47), cowplot v.1.1.1 (48), readxl v.1.3.1 (49), ape v.5.5 (39), and pheatmap v.1.0.12 (50).

### Human subjects research.

The study was reviewed and approved by the Tennessee Department of Health institutional review board (IRB) (see 45 CFR part 46; 21 CFR part 56). The Minnesota and Connecticut Department of Health IRB determined that the study was exempt from IRB review in accordance with 45 CFR 46.101(b)(4). The study was approved by the CDC IRB with a waiver of HIPAA authorization under the Privacy Rule as per 45 CFR 46.512(i). The University of Michigan Medical School IRB approved this protocol.

### Data availability.

Study isolates included isolates from BioProject no. PRJNA272863 (7) and PRJNA873034 (new from this study) (supplemental material) (51). The code and non-PHI data used to perform analyses can be found on GitHub at https://github.com/Snitkin-Lab-Umich/eip-cre-transmission-ms.

## RESULTS

Over 90% of collected clinical KPC-CRE isolates were KPC-Kp or KPC-Eh; we therefore focused our analysis on this subset of isolates to investigate the pathways leading to the dominant causes of KPC-CRE infections across the three study regions. We further restricted our analysis to remove redundant isolates from the same patients by including only the first isolate of each unique ST-facility-patient combination, yielding 255 isolates. In each state, over 50 KPC-Kp and KPC-Eh isolates were present across over 20 distinct health care facilities (see Table S1 and Fig. S1A and B in the supplemental material). The number of distinct STs varied by state (Fig. S1C), with the dominant STs being KPC-Kp ST258 in all three states, KPC-Eh ST171 in Minnesota, and KPC-Eh ST114 and KPC-Kp ST307 in Tennessee.

### Clonal dissemination after importation or *bla*_KPC_ acquisition differs across states and STs.

**FIG 1:**
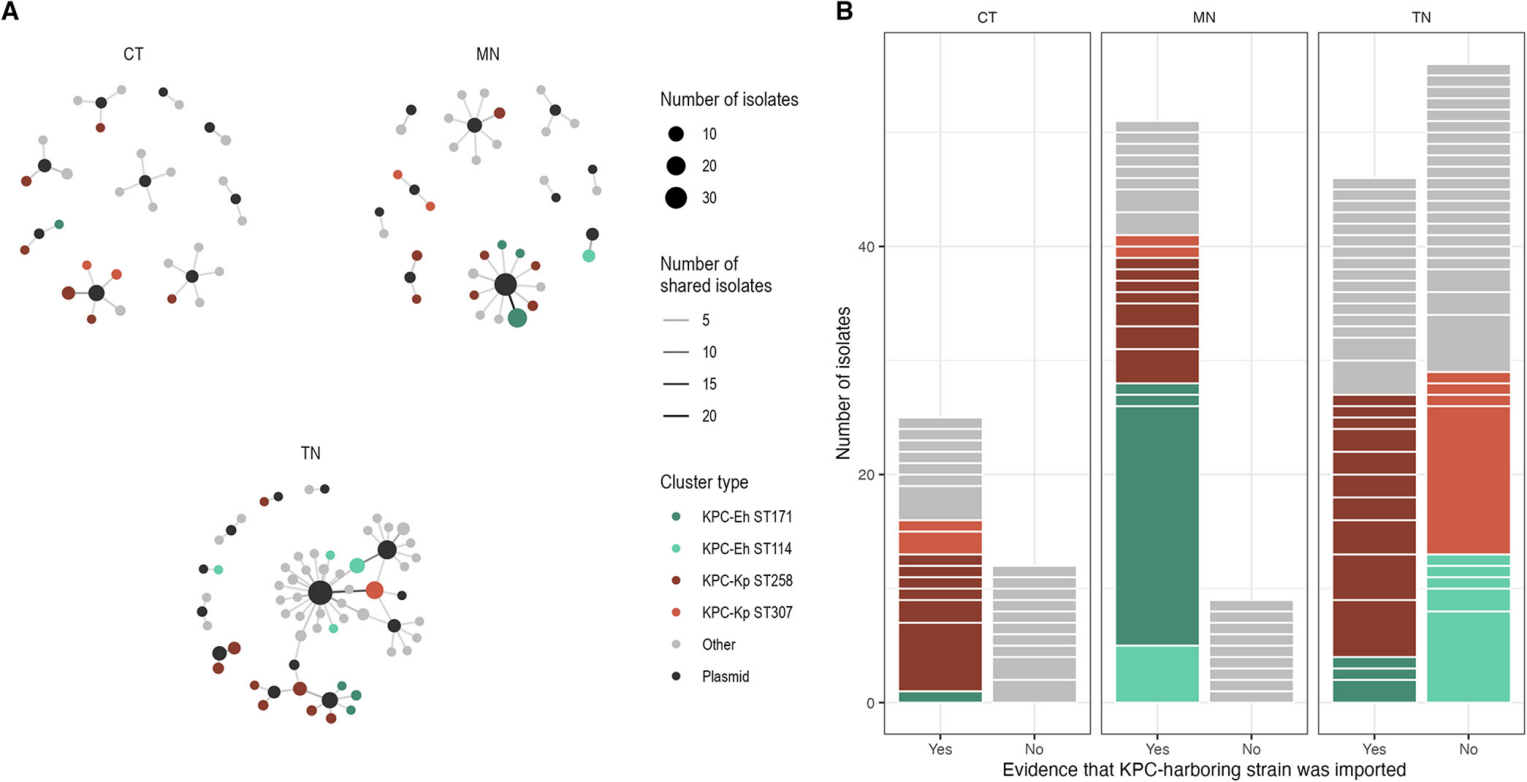
Evidence of importation and clonal dissemination differs across states and sequence types (STs). Phylogenetic clusters containing isolates from each state were identified using publicly available genomes to provide genetic and geographic context and extracting subclades that were monophyletic for a single state. MOB-suite and blastn were used to predict *bla*_KPC_ plasmids carried by isolates in each region. (A) Bipartite graphs show which plasmids (black circles) are predicted to be harbored by isolates in each phylogenetic cluster (colored/gray circles). Most phylogenetic clusters are associated with a single plasmid, with the exception being 5 clusters in Tennessee, which were presumed to derive from multiple plasmid acquisition events into common strain backgrounds. (B) Phylogenetic clusters are separated by white lines between the colored bars, where the bar height of a given cluster is the cluster size. Importation events were considered those where genetic neighbors of a phylogenetic cluster from other states were already associated with *bla*_KPC_ (see Materials and Methods). We observed events with limited onward transmission in all states. We also see a large cluster of *E. hormaechei* ST171 in Minnesota that arose from a putative importation event, indicating importation followed by sustained dissemination. Additionally, we observed large clusters of *E. hormaechei* ST114 and K. pneumoniae ST307 in Tennessee with no evidence of importation from another state. ST, sequence type; Eh, *E. hormaechei*; Kp, K. pneumoniae.

To gain insight into the origin and magnitude of spread of different KPC-Kp and KPC-Eh strains in each state we performed phylogenetic analyses, including 5,346 publicly available Enterobacter and Klebsiella species genomes deposited in the PATRIC database (Fig. S2A and B). We sought to use the genetic and geographic context provided by PATRIC isolates to identify local phylogenetic clusters that included only isolates from a given state (Fig. S2C), with each cluster presumed to derive from a single importation or local plasmid transfer event. To distinguish between a cluster deriving from importation or local plasmid transfer, we considered whether genetic neighbors of the cluster from other states harbored *bla*_KPC_ (see Materials and Methods). Consistent with these phylogenetic clusters deriving from single importation or plasmid transfer events, we observed that phylogenetic clusters including more than one isolate contained the same KPC-associated plasmid in 22/27 (81.5%) cases. The 5 clusters where isolates contained multiple KPC-associated plasmids all occurred in Tennessee (Fig. 1A; Fig. S2D), 4 of which were inferred to derive from local plasmid transfer based on genetic neighbors from other states being KPC negative. Thus, these 4 Tennessee phylogenetic clusters likely derived from multiple plasmid acquisition events on a common genetic background within the region.

Despite what we assume to be relatively sparse sampling of circulating Klebsiella and Enterobacter strains in PATRIC, phylogenetic and plasmid context revealed that KPC-Kp and KPC-Eh cases in all three states were attributable to large numbers of independent regional emergence events. In Connecticut and Minnesota, large phylogenetic clusters consistent with local dissemination were almost exclusively restricted to those that could be traced back to the outside importation of the epidemic lineages KPC-Kp ST258 and KPC-Eh ST171. In Tennessee, we also detected evidence for local dissemination of imported KPC-Kp ST258 and, to a lesser extent, KPC-Eh ST171. However, in contrast to Connecticut and Minnesota, in Tennessee we also found large phylogenetic clusters that could not be traced to an external source. Most notable were large clusters of the emerging epidemic clones KPC-Kp ST307 (52) and KPC-Eh ST114 (53). Support for these phylogenetic clusters being due to local acquisition of *bla*_KPC_ comes from the nearest neighbors of these strains in public collections being KPC negative (Fig. S2C) and their harboring a *bla*_KPC_ associated plasmid shared by many KPC-Kp and KPC-Eh strains in Tennessee (see below).

### Genomic analysis of regional clinical isolate collections allowed for the detection of local transmission in Minnesota and Tennessee and lack thereof in Connecticut.

Having characterized the origin of circulating strains in each region, we next sought to understand whether regional genomic surveillance of clinical isolates was dense enough to discern facilities and regional subnetworks where transmission was occurring. To this end, we first investigated the extent to which the surveillance isolates captured putative recent local transmission events, defined as small pairwise SNV distances (sensitivity analysis with ≤5, 10, or 15 SNVs) (Fig. S3B) or small patristic distances (Fig. S3C) (54). We captured very few local transmission events in Connecticut, suggesting that importation from other geographic regions may be driving the case load there. In contrast, we captured likely local transmission events of KPC-Eh ST171 in Minnesota and of KPC-Kp ST258, KPC-Kp ST307, and KPC-Eh ST114 in Tennessee across all SNV thresholds, consistent with our observation of large phylogenetic clusters for these STs (Fig. 1).

**FIG 2:**
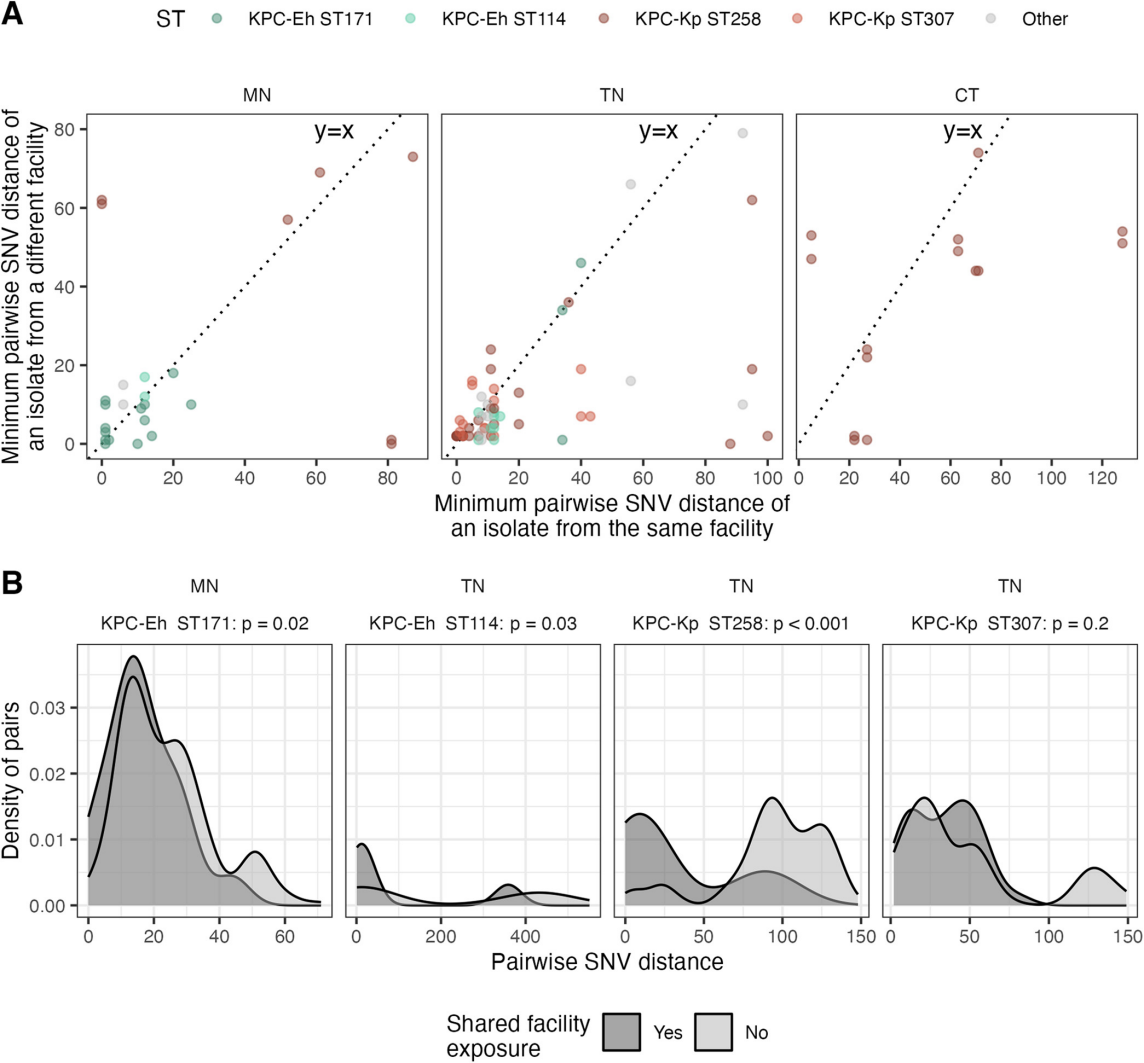
Small pairwise single nucleotide variant (SNV) distances between isolates suggest that local transmission was captured. (A) For each isolate with at least one intrafacility isolate pair and at least one interfacility isolate pair, pairwise SNV distance to the most closely related isolates from the same or different facilities is shown. (B) Pairwise SNV distance of interfacility isolate pairs from patients with a shared facility exposure compared to those not linked by a shared facility exposure. A shared facility exposure is when individuals with isolates from different facilities both spent time in the same facility at some point in the last year. Only STs with >10 isolate pairs with a pairwise SNV distance of ≤15 SNVs are shown. One-sided Wilcox *P* values compare pairwise SNV distances of isolates with a shared facility exposure (*n* ranges from 14 to 35) to isolates without a shared facility exposure (*n* ranges from 67 to 376). SNV, single nucleotide variant; ST, sequence type; Eh, *E. hormaechei*; Kp, K. pneumoniae.

Examination of isolate pairs linked by close genetic distances revealed that they come from both patients at the same facility and those at different health care facilities (Fig. 2A). Of note, while there was no enrichment in closely related isolates among patients whose isolates were collected at the same facility, there was a significant enrichment in close genetic distances for patients who share an exposure to any facility (Fig. 2B). Detection of intrafacility transmission clusters was not enhanced by considering additional epidemiologic data, as patients with closely related intrafacility isolate pairs did not share any underlying conditions that may be indicative of an outbreak related to a certain location or procedure in the hospital (all *P* values of >0.05). This suggests that while restriction to clinical isolates may hinder detection of intrafacility transmission clusters, signatures of regional transmission can be identified by considering patient sharing between health care facilities.

### Longitudinal genomic surveillance in Minnesota revealed limited spread of ST258 and localized transmission of ST171 in a regional subnetwork.

**FIG 3:**
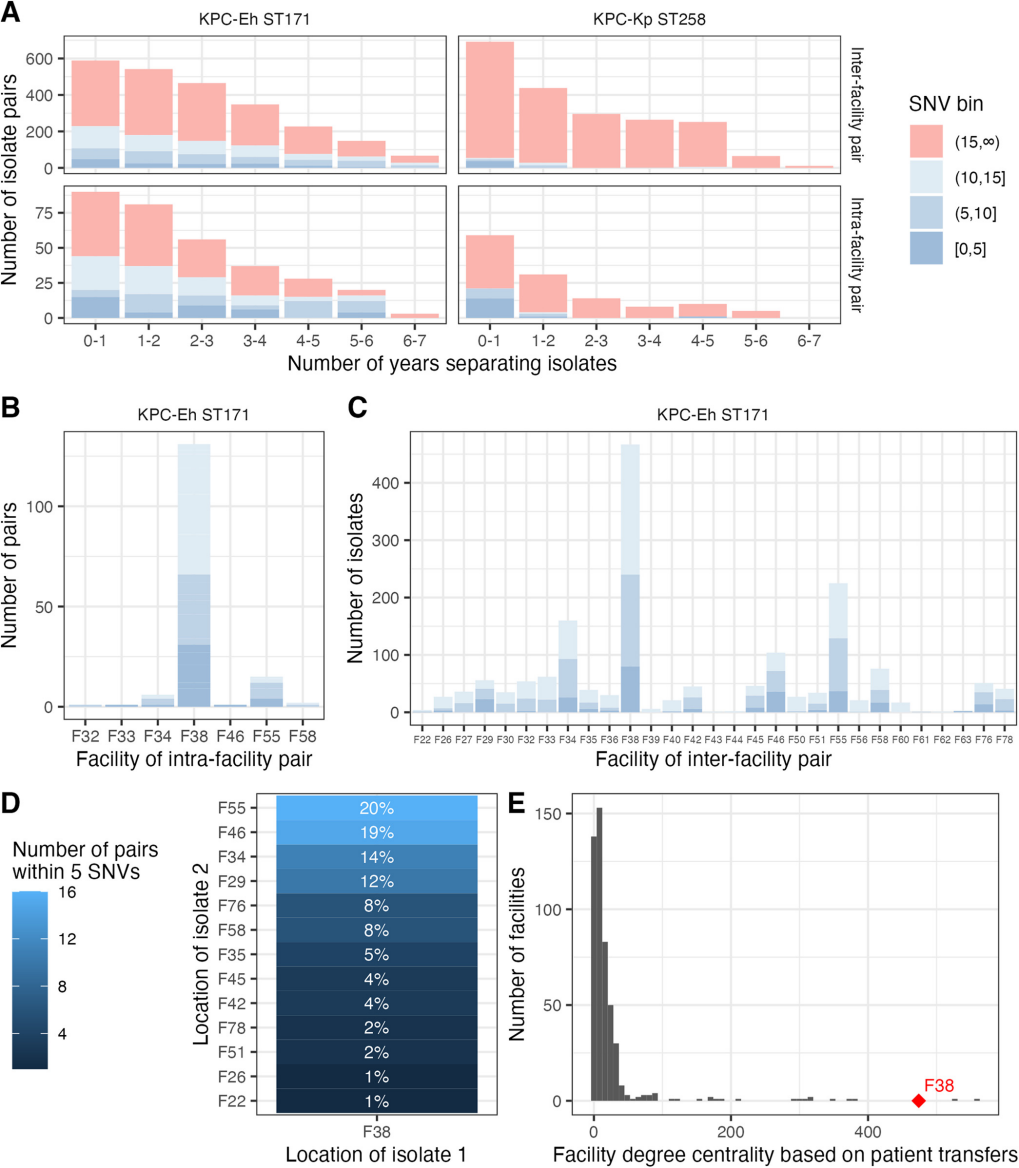
Transmission of KPC-Eh ST171 in Minnesota. (A) The number of years separating ST171 and ST258 isolate pairs, stratified by the genetic distance between strains, shows that closely related ST171 isolates are observed years apart. (B) Grouping of intrafacility ST171 isolate pairs by facility show that most pairs are from facility F38. (C) Grouping of interfacility isolate pairs by facility again shows facility F38 being overrepresented in interfacility pairs with small genetic distances. (D) Facilities sharing ST171 isolates within 5 SNVs of isolates from facility F38 show many connections with a small number of satellite facilities. (E) Degree centrality of all facilities, with F38 highlighted as among the most connected facilities in Minnesota. Degree centrality of a given facility is defined as the number of facilities connected to that facility by at least one patient transfer in the past year. The in- and out-degree values were summed (patient transfers from and to a facility). The values to and from each facility were summed. SNV, single nucleotide variant; ST, sequence type; Eh, *E. hormaechei*; Kp, K. pneumoniae.

With evidence for isolate pairs with small SNV distances being informative of recent regional transmission, we next set out to look at genomic linkages across Minnesota and Tennessee to gain insight into where transmission of different STs was occurring. Minnesota has been collecting KPC-CRE isolates since 2012. Incorporating information from this extended data set to investigate transmission, we observed stark contrasts in the relatedness of ST171 compared to ST258 isolates across time. ST258 isolates collected over 1 year apart were rarely closely related to each other; however, ST171 isolates collected even over 5 years apart were often quite closely related (Fig. 3A), suggesting a persistent local reservoir for ST171. We investigated whether these closely related ST171 pairs were centered around specific facilities and found that one facility, F38, was highly represented in closely related ST171 intrafacility (Fig. 3B) and interfacility (Fig. 3C) isolate pairs. Of all ST171 isolate pairs within 5 SNVs, 31/38 (82%) intrafacility pairs were from F38 and 80/136 (59%) interfacility pairs contained one isolate from F38. About half of these interfacility pairs were connected to one of three other facilities (Fig. 3D). F38 is overall highly connected to other facilities in the patient transfer network (Fig. 3E) and is particularly well connected by patient transfer to the other three facilities, with many close ST171 genetic linkages (≥90th percentile of all facility pairs). Combined with the phylogenetic cluster analysis above, these findings indicate that while ST258 detection over time is due to frequent reintroduction, ST171 is maintained by entrenchment in a local facility subnetwork.

### Distinct facility subnetworks in Tennessee harbor different lineages.

**FIG 4:**
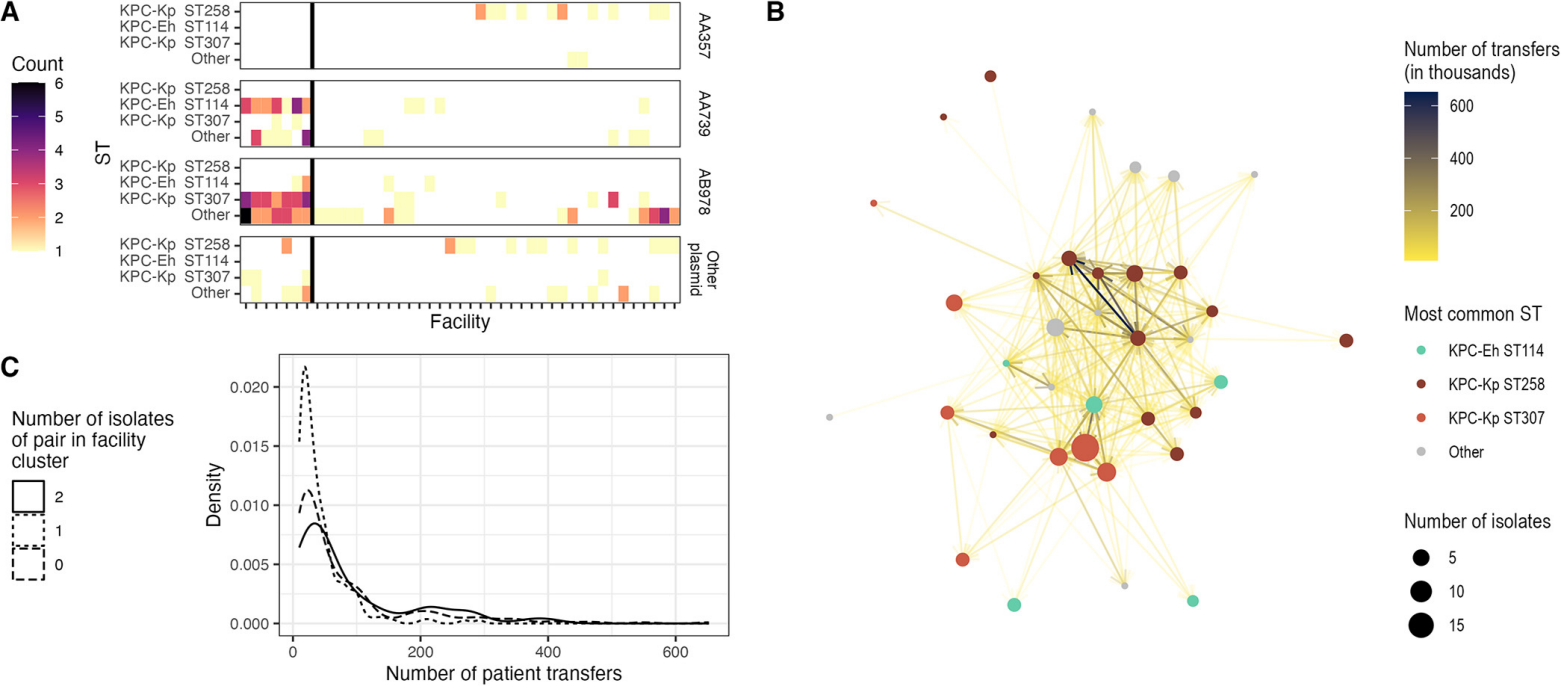
Distinct facility subnetworks in Tennessee harbor different lineages. (A) The number of patient exposures in the prior year to each facility is shown for each ST and plasmid. Each patient may be represented more than once if they have more than one facility exposure. A cluster of facilities identified using the complete linkage clustering method (left of the solid black line) are common in patients with ST114, ST307, and other isolates and often contain plasmid cluster AA739 or AB978. (B) Patient transfer network of facilities with at least one whole-genome-sequenced isolate plotted using the Kamada-Kawai algorithm. Facilities where ST114 or ST307 are most common are clustered in the network. (C) The numbers of patient transfers for facility pairs where 0, 1, or 2 of the facilities are ones where ST114 or ST307 are most common. Facility pairs where ST114 or ST307 are most common in both have more patient sharing than facility pairs where ST114 or ST307 is most common in 0 or 1 of the facilities. ST, sequence type; Eh, *E. hormaechei*; Kp, K. pneumoniae.

We next explored whether we could discern where transmission was occurring in Tennessee by overlaying genomic data on health care facility networks. We observed that even at the level of ST, health care facilities showed distinct profiles—those with primarily ST258, those with only rare STs, and those with a mix of ST114, ST307, and rare STs (Fig. 4A). To test the hypothesis that facilities showing mixtures of STs could be sites of *bla*_KPC_ plasmid transfer, we examined plasmid profiles of isolates across facilities. Strikingly, the isolates at facilities with mixed STs generally contained one of two KPC-associated plasmids, one of which was carried by ST114 and the other by ST307 (Fig. 4A; Fig. S5). In contrast, there was little evidence of plasmid sharing involving ST258 and other isolates from facilities where ST258 was detected. Visualization of facilities where ST307 and ST114 are dominant in the context of the aggregate patient transfer network revealed that they are closely connected (Fig. 4B) and have more patient sharing among themselves than with other facilities (Fig. 4C) (Wilcox test; *P* < 0.001). Together, these findings suggest the diversity of STs harboring *bla_KPC_* in these facilities is being driven by frequent plasmid transfer and subsequent spread of nascent KPC-Kp and KPC-Eh strains between health care facilities via patient transfer.

## DISCUSSION

As public health laboratories increase their capacity for genomic surveillance, it is critical to understand if and how genomic data from passive surveillance can be used to guide regional intervention efforts. Statewide active surveillance is infeasible, and while that would be required to infer who transmitted to whom, passive surveillance may be enough to provide practical guidance for interventions, including if and where transmission is happening and if there are any emerging threats. Here, we found that genomic analysis of clinical *bla*_KPC_-positive CRE isolates from three U.S. states can inform our understanding of the origins and transmission pathways of circulating strains.

We could discern the relative contributions of importation and clonal dissemination to each state’s KPC-Kp and KPC-Eh case burden and highlight individual facilities and connected regional subnetworks where putative transmission occurred. Importantly, these insights could not have been made using PCR screening, as data from PCR cannot be used to contextualize isolates from common strains and determine whether and where transmission is occurring. Furthermore, these insights did not rely on detailed clinical metadata, indicating that other regions interested in identifying hot spots and pathways for regional spread may be able to do so with only WGS and knowledge of recent health care exposures. Our findings suggest that the increased analytic capacity required for genomic analysis of clinical isolates at SPHLs may be worth prioritizing to be able to monitor transmission hot spots, as insights gleaned from these investigations may enable targeting of regional infection prevention efforts to certain facilities.

Despite focusing on the same two *Enterobacterales* species harboring the same carbapenemase gene, we observed stark differences in the underlying drivers of each state’s KPC-Kp and KPC-Eh burden. In Connecticut, strains were frequently imported but did not show evidence of onward transmission. Importation is unsurprising, given the state’s proximity to other major CRE hot spots in the Northeast United States (55). However, this still leaves unanswered why imported *bla*_KPC_-positive CRE have apparently not gained a foothold. One reason may be that the structure of the Connecticut regional health care network decreases local transmission. Recent work showed that post-acute care settings can act as initiators and amplifiers of regional CRE epidemics (56, 57). Thus, infection prevention practices in these facilities and their patient transfer connections with other facilities may have large impacts on overall regional CRE spread. Regardless, our observations suggest that focusing resources on testing admission cultures from out-of-state may allow Connecticut to continue having a low burden of these CRE strains.

In Minnesota, *bla*_KPC_-positive CRE burden was driven by a major importation event of the epidemic lineage KPC-Eh ST171, followed by dissemination within the region. Incorporation of historical data revealed that isolates far apart in time were often closely related and were largely from or connected to isolates from a single hub acute care facility with many referrals that provides specialty care, F38. These observations could indicate an environmental reservoir within or proximate to F38 or undetected endemic spread at one or more referral facilities. Having these data in real time would generate actionable hypotheses to potentially reduce regional spread. F38 could prioritize testing patients admitted from facilities connected by extensive patient transfer. Similarly, state epidemiologists could investigate facilities with large numbers of genomic linkages to isolates from F38, such as the three identified here, to detect other potential reservoirs and locations where intervention may have a broad positive impact on regional KPC-Eh burden.

Tennessee showed a diversity in circulating strains. In addition to a significant burden of ST258, we observed CRE isolates from two more recently emerged lineages—KPC-Kp ST307 and KPC-Eh ST114—and less-common STs that appear to have emerged and spread more recently via two KPC-associated plasmids. This pattern is in stark contrast with Connecticut and Minnesota, where there were very few cases of onward transmission detected after a nonepidemic strain acquired *bla*_KPC_. These observations suggest that the specific plasmids harboring *bla*_KPC_ in a region, as well as the strains they are carried by, may dictate the risk for plasmid transfer and the capacity for strains acquiring these plasmids to spread. The importance of existing strain-plasmid combinations in the emergence of new strains is further illustrated by our observation that facilities in Tennessee where ST258 KPC-Kp was most common showed little evidence of plasmid transfer or spread of nascent CRE-KPC strains. While we were unable to discern specific HGT events in this study due to our limited sampling (i.e., the isolate donors and recipients of plasmids), our observations do suggest that in addition to detecting clonal spread of known epidemic threats, regional genomic surveillance can detect high-risk plasmids and the facilities and subnetworks where they are spreading.

Our study has several limitations. First, our use of only clinical isolates may lead to an underestimation of clonal dissemination, which may be different for different STs, depending on their virulence and associated colonization-to-infection ratio (i.e., ST-specific iceberg effect). Additionally, lack of environmental isolates precluded us from identifying potential environmental reservoirs contributing to transmission. However, we were still able to uncover patterns of clonal dissemination using these data, suggesting that regional clinical isolate collections can provide actionable insights for public health labs. While transmission may not have occurred at the facilities where clinical isolates were collected, this nevertheless provides a location at which to initiate an investigation and could ultimately guide more targeted surveillance efforts. Second, the isolate collection periods were different for each state. This may result in different levels of contextual information provided by public databases, through which importation was inferred. However, our observations are consistent with prior studies in that KPC is known to be stably associated with ST258, ST171 emerged in the Upper Midwest, and ST307 and ST114 only recently became associated with KPC. Another limitation is that the public isolates used from PATRIC to inform the origin of locally circulating strains, despite being curated, are subject to errors and data quality issues. Additionally, they are not equally representative of all geographic regions, which may underestimate the extent of importation as multiple importations from an unsampled reservoir may be merged. While the analyses performed here would be enhanced by cleaner data and additional context, we note that inferences made into importation using phylogenetic clustering are largely consistent with the SNV distance-based analyses, supporting the robustness of our conclusions. Furthermore, the aggregate patient transfer data used to understand the connectivity between regional health care facilities was derived from Medicare patient transfers, which may lead to biases in patient transfer connections. However, despite this, we were able to discern local subnetworks of health care facilities with distinct transmission patterns. Finally, we only performed short-read sequencing and were therefore unable to thoroughly dissect plasmid structural variation and transposon hopping between plasmids. However, by comparing short-read data to databases of sequenced plasmids, we were able to identify predicted plasmid sharing between strains, with putative plasmid transfer events being strongly supported by their enrichment within facilities and connected subnetworks.

In conclusion, our results suggest that the origins and transmission patterns of *bla*_KPC_-positive CRE can be investigated using genomic surveillance of sufficiently comprehensive regional clinical isolate collections and that these transmission dynamics can vary across strains and regions. The differences underlying the KPC-Kp and KPC-Eh burdens in these three states would not have been discernible without genomic data and in fact would be strengthened further by the existence of larger genomic surveillance initiatives that could provide more granular context for the origin of strains and their mobile elements to guide prevention efforts. Similarly, our understanding of how KPC-Kp and KPC-Eh proliferate in a region would be limited without knowledge of patient health care exposures and transfer networks, which enable a more nuanced understanding of where transmission is occurring, where reservoirs might exist, and where additional surveillance or intervention is required. Taken together, these observations support the value of robust regional genomic surveillance for antibiotic resistance threats and the need for analytic platforms capable of integrating genomic and patient movement data to guide local and state infection prevention efforts in real time. Future work should investigate the impact of interventions informed by real-time genomic analysis.
