# Supplementary material for: Distinct Origins and Transmission Pathways of blaKPC Enterobacterales across Three U.S. States
Source: J Clin Microbiol. 2023 Jul 13;61(8):e00259-23. doi: 10.1128/jcm.00259-23 (PMC10446861; doi:10.1128/jcm.00259-23)
Supplement: Supplemental file 1 — Supplemental methods, Table S1, and Fig. S1 to S5. Download jcm.00259-23-s0001.pdf, PDF file, 1.7 MB [file jcm.00259-23-s0001.pdf]

Supplemental information

Supplemental methods

State-specific CRE surveillance methods

While all CRE are supposed to be tested for carbapenemases, this does not always happen in practice; the percentage of CRE tested for carbapenemases was 81.5% in CT, 90% in MN, and 78% in TN.

Connecticut

CT did not participate in MuGSI surveillance during the study period, so all study isolates from CT came from statewide passive surveillance for CRE. CRE surveillance of all Enterobacterales isolates collected from all invasive clinical sites, respiratory sources, and urine began on Jan 1, 2017. All CRE isolates undergo antimicrobial susceptibility testing with a custom Sensititre and disk diffusion panel, phenotypic detection of carbapenemase activity using the mCIM test and PCR detection of carbapenemase genes (bla_{KPC}, bla_{NDM}, $bla_{\text{OXA-48}}$, bla_{VIM}, bla_{IMP}). Isolates carrying bla_{KPC} that were identified in 2017 and 2018 from this collection were included.

Minnesota

MuGSI surveillance in Minnesota is conducted in Ramsey and Hennepin counties. MuGSI isolates are obtained from sterile sites or urine and include *E. coli*, *Klebsiella* spp., and *Enterobacter* spp. Non-MuGSI isolates are collected from a variety of culture sources (e.g., blood, urine, respiratory) and represent multiple Enterobacterales species. Isolate identification is confirmed by the MALDI Biotyper CA System (Bruker Daltonics, Inc., Billerica, MA). All CRE

isolates are tested for carbapenemase production using the modified carbapenem inactivation method (mCIM). Isolates demonstrating phenotypic carbapenemase production undergo molecular testing (i.e., polymerase chain reaction [PCR]) for detection of carbapenemase genes *bla*$_{KPC}$, *bla*$_{NDM}$, *bla*$_{OXA-48}$, *bla*$_{VIM}$, *bla*$_{IMP}$. All *bla*$_{KPC}$ -positive *K. pneumoniae* and *E. cloacae* complex isolates submitted to MN PHL from 2012-2018 were analyzed for this study.

Tennessee

EMS region 2-7 were selected because it provides a good representation of patient referrals; captures most isolates in patient transfer networks covering the area; and includes both the Knoxville area in which CRE emerged in 2016, and the Nashville area (the MuGSI catchment area: Dickson, Cheatham, Robertson, Sumner, Wilson, Rutherford, Williamson and Davidson counties) where CRE increased in 2017. CRE isolates underwent organism confirmation using the MALDI-TOF bioMerieux. Isolates were tested for antimicrobial resistance with the Kirby Bauer method. Those isolates that were resistant to at least one carbapenem were then tested by the CarbaNP Method for carbapenemase production. Those that demonstrated carbapenemase production from the CarbaNP underwent a PCR assay developed by the CDC to test for the *bla*$_{KPC}$ and *bla*$_{NDM}$. All *bla*$_{KPC}$-positive isolates collected during 2016–2017 and submitted to the TN SPHL were included.

Epidemiologic data

MuGSI surveillance data were collected through medical record review by EIP surveillance officers in each site. Additional details on data collection for isolates identified outside of the MuGSI program are listed below for each site.

Connecticut

Clinical case details were abstracted from medical records by EIP surveillance officers with the Connecticut Department of Public Health.

Minnesota

At the time of culture collection, surveillance staff completed CRF through electronic or in-person medical chart review.

Tennessee

For all carbapenemase-producing Enterobacterales cases, a questionnaire is completed by the healthcare facility of origin. For cases that did not have a completed questionnaire, hospitalization details were gathered from the Statewide Hospital Discharge data (described below).

<u>Facility identification</u>

Facility identification was performed through chart review at the reporting facility. State-specific details are described below.

Connecticut

Chart review was performed at the reporting facility for all inpatient and hospital network events. When additional referring facilities (including nursing homes, long term acute care hospitals, etc.) were identified on medical record review, follow-up investigation was performed at the

additional facilities. Upon identification of outpatient isolates, patient details were collected from ordering providers by phone call and/or faxed data collection instrument.

Minnesota

MN staff maintain a compendium with facility name and type according to CMS. The facility name and type are recorded in the MN surveillance database for each isolate and verified using this compendium. For MuGSI cases, chart review at the facility where the isolate was collected was also performed by MN staff.

Tennessee

TN staff linked patient identifiers of each case in the National Electronic Disease Surveillance System (NEDDS)-Base System (NBS) surveillance data to the TN Hospital Discharge Data System (HDDS) to identify and confirm the healthcare facility where they had the first specimen culture collected for each CP-CRE case in TN. Alternatively, if the patient was not hospitalized in a TDH-licensed hospital and therefore, was not found in the HDDS, TN staff used surveillance data from NBS and the afore-mentioned questionnaire to identify the healthcare facility of origin. Using this approach, the healthcare facility of origin was identified for 139 (89%) of the 157 included isolates. TN staff also gathered information on healthcare exposures within one year prior to specimen collection by linking the patient identifiers in the surveillance data with the inpatient and outpatient HDDS dataset. Isolates with no healthcare exposure information were not used for the patient sharing network analysis (see Methods).

Linkage/matching of facility names to CMS ID

Linkage of facility names to CMS ID was performed at each respective SPHL.

Connecticut

Facilities were matched with CMS IDs using CMS Provider of Services Files available at: https://www.cms.gov/Research-Statistics-Data-and-Systems/Downloadable-Public-Use-Files/Provider-of-Services

Minnesota

In MN, CMS IDs were manually entered for each isolate using Excel.

Tennessee

In TN, the name healthcare facility of origin was linked with the registered facility name by CMS ID using the SPEDIS and COMPGED commands in SAS 9.4 (SAS Institute, Cary, North Carolina) to allow for inexact matches that accounted for minor spelling variations between the facility names from TN's surveillance data and those from the CMS dataset. Due to the data-sharing agreement between TDH and the University of Michigan, the facility names and CMS IDs in the WGS dataset and patient transfer data from CMS were de-identified before sharing with The University of Michigan.

<u>Generation of aggregate patient transfer networks</u>

Aggregate patient transfer networks for each state for the year 2017 were derived from Centers for Medicare and Medicaid Services (CMS) fee for service beneficiary claims data linked to the CMS Minimum Data Set (MDS) by Medicare beneficiary ID. The dataset comprises a network

of medical facilities, including but not limited to short-term acute care hospitals, skilled nursing facilities and long-term acute care hospitals, but may not be representative of non-Medicare beneficiaries. To create an annual transfer network, CDC staff linked each unique beneficiary's healthcare utilization. This included CMS inpatient claims for acute care stays and the MDS to ascertain a beneficiary's presence in a nursing home, regardless of payer for that stay. Transfer networks were comprised of the year of interest and looked back to the prior year to obtain all discharging facilities where patients had an opportunity to acquire CRE.

Transfers are defined as a patient discharged from a facility and then subsequently admitted to a facility. The number of transfers between two facilities includes transfers directly from one facility to another as well as transfers with an intervening stay in the community of less than 365 days. We chose to use a 365-day time interval rather than a smaller time interval because this gives us more resolution in comparing the extent of patient flow between facilities. Any facility pairs with 10 or fewer patient transfers were censored, as per data use agreements between CDC and CMS.

For each state, we subset the aggregate patient transfer network to only include the facilities for which we have at least one WGS isolate or ones that are connected to one of these facilities by at least one patient transfer event over the course of a year. We then identified paths of maximum patient flow between this subset of facilities using the get_patient_flow() function in regentrans v0.1 [37].

DNA extraction

Connecticut

Total DNA extractions performed by CT DPHL for WGS were either by the manual method or automated on the QIAcube. Manual extractions from pure single colony Gram-negative bacterial isolates by QIAgen DNeasy Blood and Tissue kit and follow-up quality control of all purified extracts were conducted following PulseNet Protocols for Whole Genome Sequencing on the Illumina MiSeq system. Automated extractions of bacterial cell pellets were performed using the QIAcube protocol for Isolation of DNA from Gram-negative bacteria and QIAGEN DNeasy Blood and Tissue kit. To obtain the cell pellets, colonies from overnight plates were resuspended in 1x Phosphate buffered saline (PBS, Sigma) to an OD600 of 0.35-0.40. Two milliliter cell suspension were centrifuged at 5,000 *x g* for 5 minutes at 2-8 °C and used for extractions.

Minnesota

DNA extraction of the isolates was performed in MN using either the QIAcube (Qiagen, Germantown, MD) extractor and QIAmp DNA Mini extraction kit (Qiagen, Germantown, MD) or the Roche MagnaPure LC and the TNA Isolation extraction kit.

Tennessee

DNA extraction of the isolates was performed in TN on a Thermo Fisher, KingFisher Flex auto-extraction instrument, using an Applied Biosystems MagMAX DNA Multi-Sample Ultra extraction kit.

<u>Whole-genome sequencing (WGS)</u>

The extracted DNA was prepared for WGS using Illumina Nextera XT library Prep kits with Illumina, Nextera XT Index kits (MN, TN, CT) or with Illumina DNA Prep kit and Illumina CD Indexes (CT) (Illumina, San Diego, CA). The Library preparation process was performed on a Beckman Coulter Biomek Fxp instrument (Beckman Coulter, Brea, CA) (TN), on the Eppendorf epMotion (CT) (Eppendorf, Enfield, CT), or manually (MN, CT). The prepared libraries were sequenced on an Illumina MiSeq sequencing instrument (Illumina, San Diego, CA) using 2x250 (MN, TN, CT) or 2x150 (CT) paired end sequencing with V2 chemistry. The fastq files generated were transferred to the University of Michigan for processing and analysis.

Genomic data processing

All genomic data processing and analysis was performed by members of Evan Snitkin's lab at the University of Michigan.

Species/ST identification

MLST was assigned with Ariba v2.14.4 [17]. For PATRIC isolates mlst were extracted from the isolate metadata [18].

Sequence alignments

Single-nucleotide variants were called in study sequences using SNPKIT (https://github.com/Snitkin-Lab-Umich/snpkit). Quality of reads was assessed with FastQC v0.11.9 [19]. Adapter sequences and low-quality bases were removed with Trimmomatic v0.36 [20]. Variants were identified by mapping filtered reads to the KPNIH1 reference genome (GenBank accession number CP008827.1; 5,394,056 base pairs) for *K. pneumoniae* sequences

and the MNCRE9 reference genome (GenBank accession number JZDE00000000.1; 4,911,317 base pairs) for *E. hormaechei* sequences using bwa v0.7.17 [21], removing polymerase chain reaction duplicates with Picard 2.21.7 [22], removing clipped alignments using Samclip 0.4.0, and calling variants with SAMtools v1.11and bcftools [23]. Variants were filtered from raw results using GATK's VariantFiltration v3.8 (QUAL, >100; MQ, >50; ≥10 reads supporting variant; and FQ, <0.025) [25]. Consensus files generated during variant calling were recombination filtered using Gubbins v3.0.0 [24]. The alleles at each position that passed filtering were concatenated to generate a non-core variant alignment relative to the respective reference genome. Alleles that did not pass filtering were considered unknown (denoted as N in the alignment).

Reference-based phylogenies

A custom Python script was used to filter out (mask) single nucleotide variants in the whole-genome alignment that were: (i) <5 base pairs (bp) in proximity to indels that were identified by GATK HaplotypeCaller [25], (ii) in a recombinant region identified by Gubbins v3.0.0 [24], (iii) in a phage region identified by the Phaster web tool [27] or (iv) they resided in tandem repeats of length greater than 20bp as determined using the exact-tandem program in MUMmer v3.23 [26]. This whole-genome masked variant alignment was used to reconstruct a maximum likelihood phylogeny with IQ-TREE v1.6.12 using the general time reversible model GTR+G and ultrafast bootstrap with 1000 replicates (-bb 1000) [33].

Genome assemblies

Quality of reads was assessed with FastQC v0.11.9 [19]. Adapter sequences and low-quality bases were removed with Trimmomatic v0.36 [20]. The clean reads were assembled with Spades v3.14.1 (careful mode) [29]followed by Pilon v1.23 assembly correction [30]. Contigs smaller than 500bp were discarded.

Public isolates

All assemblies from the species identified within the EIP dataset were downloaded from the PATRIC database on 04/23/2021 [18]. These data were merged with assemblies from PRJNA603790, PRJNA690239, PRJNA401340, and PRJNA415194 for a total of 74,367 assemblies. Genome annotations for the dataset were generated with RAST v1.035 [31].

Core genome phylogenies

A concatenated gene alignment of core genes was generated for the entire dataset with cognac v1.0 [32]. Relevant isolates for understanding the population structure and geographic distribution of the isolates collected as part of the EIP study were identified as any isolate within 25 substitutions or the nearest phylogenetic neighbor by alignment distance. This yielded a subset of the 5,346 isolates, including study isolates and public isolates; and a second, concatenated, core gene alignment was generated with cognac for these most relevant isolates, returning the nucleotide alignment for more precise distance comparisons. To generate sequence type specific alignments, these data were divided into relevant clonal groups. Any clonal group with less than 10 members was merged into a group of its respective species. Outgroups were identified as the isolate with the lowest substitution distance to an isolate assigned to that clonal group or species, for clonal group or species alignments, respectively. A core, concatenated gene

alignment for each set of isolates corresponding to species/clonal group was generated using cognac with the corresponding outgroup specified, returning the nucleotide alignment. Recombinant positions were removed with Gubbins v3.0.0 [24]. For alignments with more than 1000 isolates included, the alignments were randomly downsampled into subsets of 250 sequences, including the outgroup in each alignment, and gubbins was run on the downsampled alignment. This was performed in triplicate, and any position identified as recombinant in the three runs was masked from the alignment. The recombination filtered alignments were then input to IQ-TREE v1.6.12 [33] to generate maximum likelihood phylogenies.

Supplemental table and figures

Table S1: Number of each facility type by state and how many facilities were represented in whole-genome sequencing (in parentheses).

Facility type	CT	MN	TN
ACH	30 (19)	50 (16)	92 (31)
LTACH	3 (0)	2 (1)	10 (4)
SNF	224 (0)	366 (5)	309 (0)
Other	7 (0)	96 (1)	48 (1)
Unknown	0 (2)	0 (12)	0 (4)

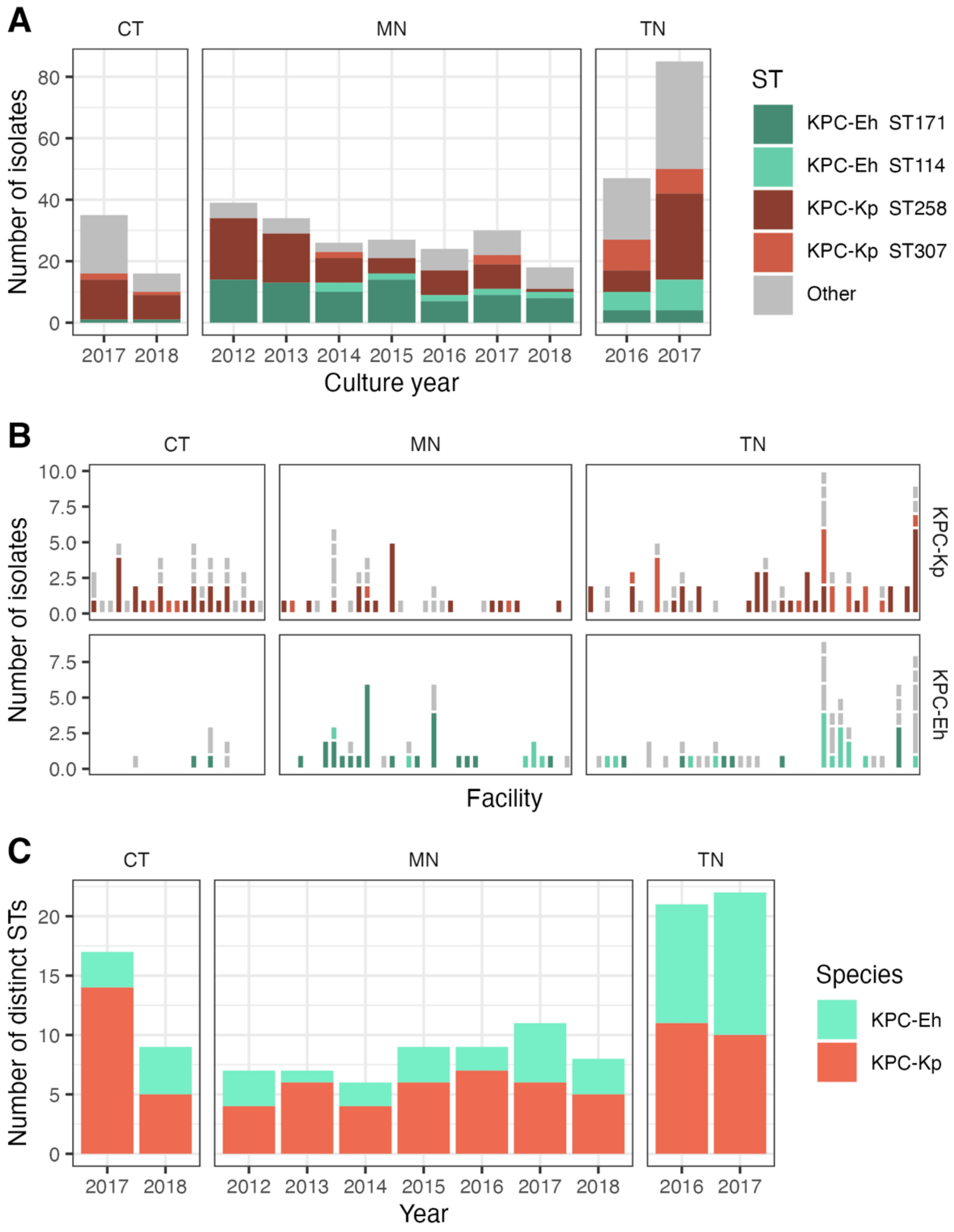

Figure S1: Distribution of species and sequence types of sequenced isolates from each state. (A) Across time. (B) Across healthcare facilities with at least one identified CRE with *bla*KPC. X axis shows facilities. Grey is any other ST of the species corresponding to that faceted plot. (C) Number of distinct STs across states and years. Notes: Only isolates from 2016-2018 were used for the majority of analyses. The entire set of MN isolates was used for the MN-specific

analysis only. The MuGSI catchment area for MN is Hennepin and Ramsey counties. Four counties in the Memphis-Delta Emergency Medical Services (EMS) and 8 in Northeastern TN were excluded due to extensive healthcare utilization across state lines; the MuGSI catchment area is TN EMS Region 5, an 8-county region encompassing the metropolitan Nashville area, of TN. CT=Connecticut; MN=Minnesota; TN=Tennessee; ST=sequence type; Eh=*E. hormaechei*; Kp=*K. pneumoniae*.

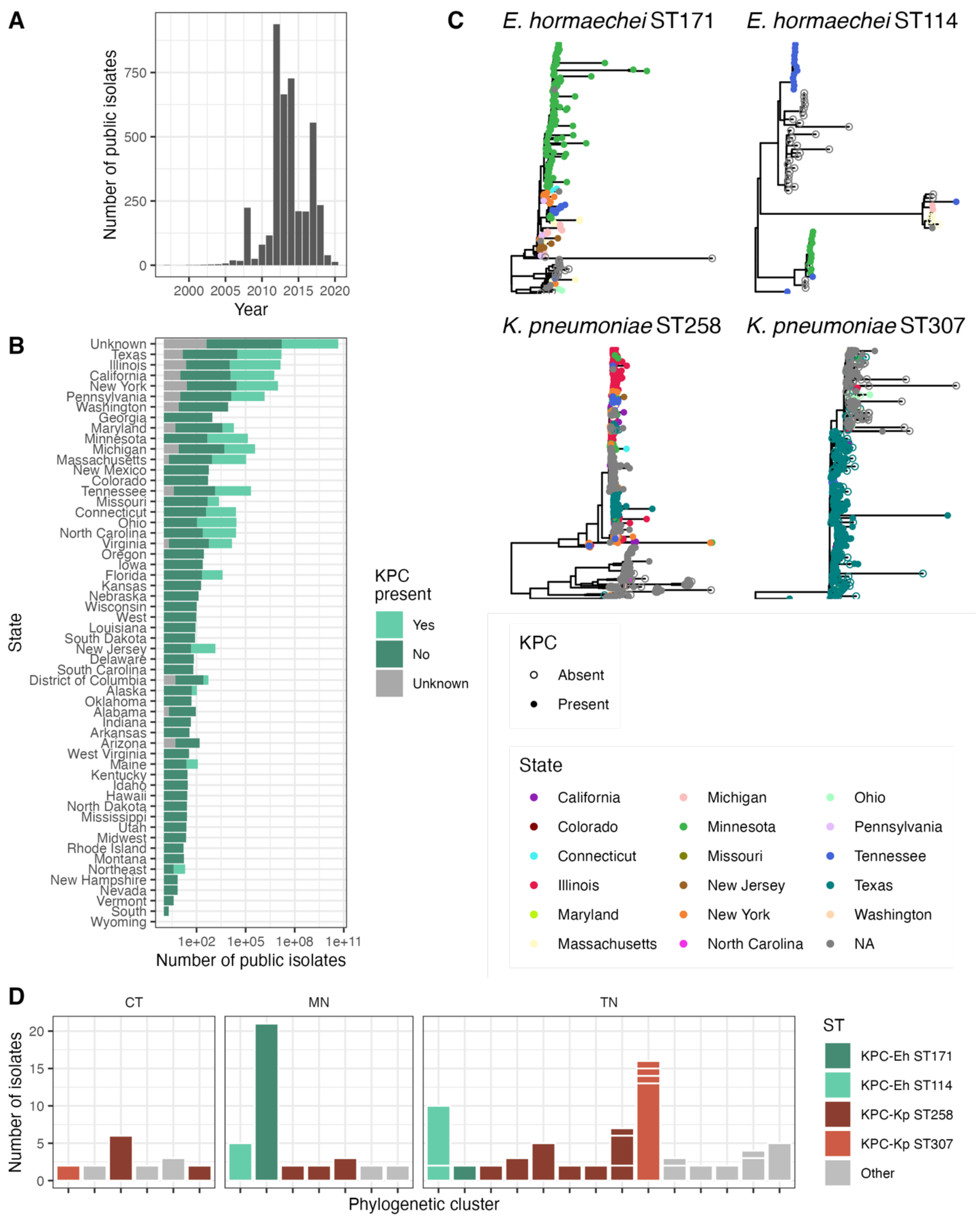

Figure S2: Information about public isolates downloaded from the PATRIC database that were included for context in the investigation of importation. (A) Distribution of year of

isolation for isolates where this information was provided. (B) Distribution of location and KPC presence/absence. (C) Phylogenies used to identify importation events, including isolates from this study. ST=sequence type. (D) Number of isolates in each phylogenetic cluster. White horizontal bars separate isolates in each cluster that contain different KPC-associated plasmid clusters.

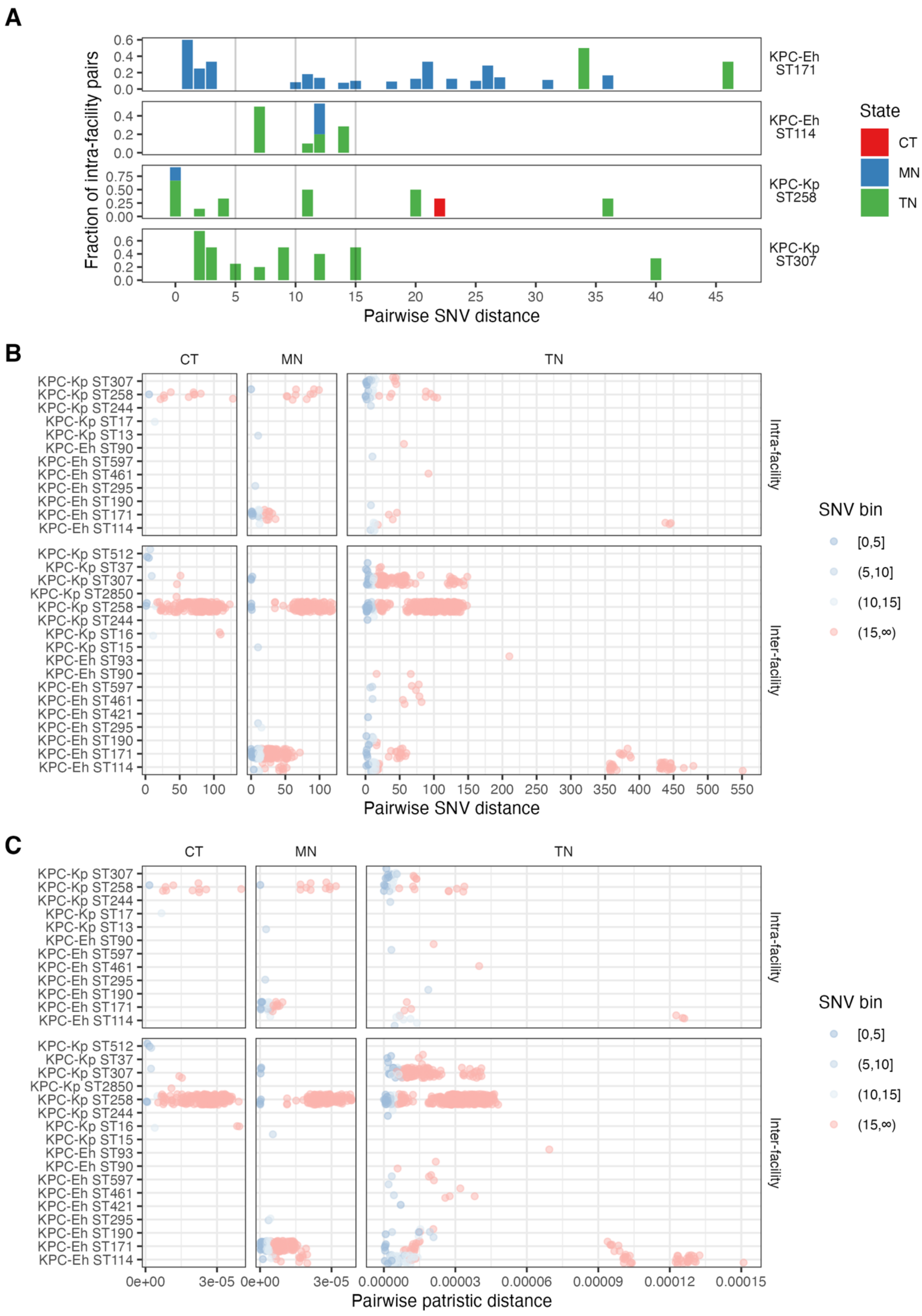
A
Fraction of intra-facility pairs
KPC-Eh ST171
KPC-Eh ST114
KPC-Kp ST258
KPC-Kp ST307
Pairwise SNV distance
State
CT
MN
TN
B
CT
MN
TN
Intra-facility
Inter-facility
SNV bin
[0,5]
(5,10]
(10,15]
(15,∞)
Pairwise SNV distance
C
Pairwise patristic distance

Figure S3: Inter-facility genomic analysis. (A) Fraction of intra-facility isolate pairs across pairwise single nucleotide variant (SNV) distances. This plot was used to identify pairwise SNV distance cutoffs based on a decrease in the proportion of intra-facility isolate pairs. Vertical lines at 5, 10, and 15 SNVs represent potential cutoffs for determining what isolates are considered closely related; 3 thresholds were chosen as a sensitivity analysis. (B) Pairwise SNV distances of all STs. Within an ST, smaller pairwise SNV distances indicate potential local transmission, while larger pairwise SNV distances are not indicative of local transmission. Varying extents of transmission were captured for different states and STs. Putative local transmission events are those in the tail of the distribution (lower pairwise SNV distances). (C) Pairwise patristic distances are concordant with pairwise SNV distance thresholds. CT=Connecticut; MN=Minnesota; TN=Tennessee; ST=sequence type; Eh=*E. hormaechei*; Kp=*K. pneumoniae*; SNV = single nucleotide variant.

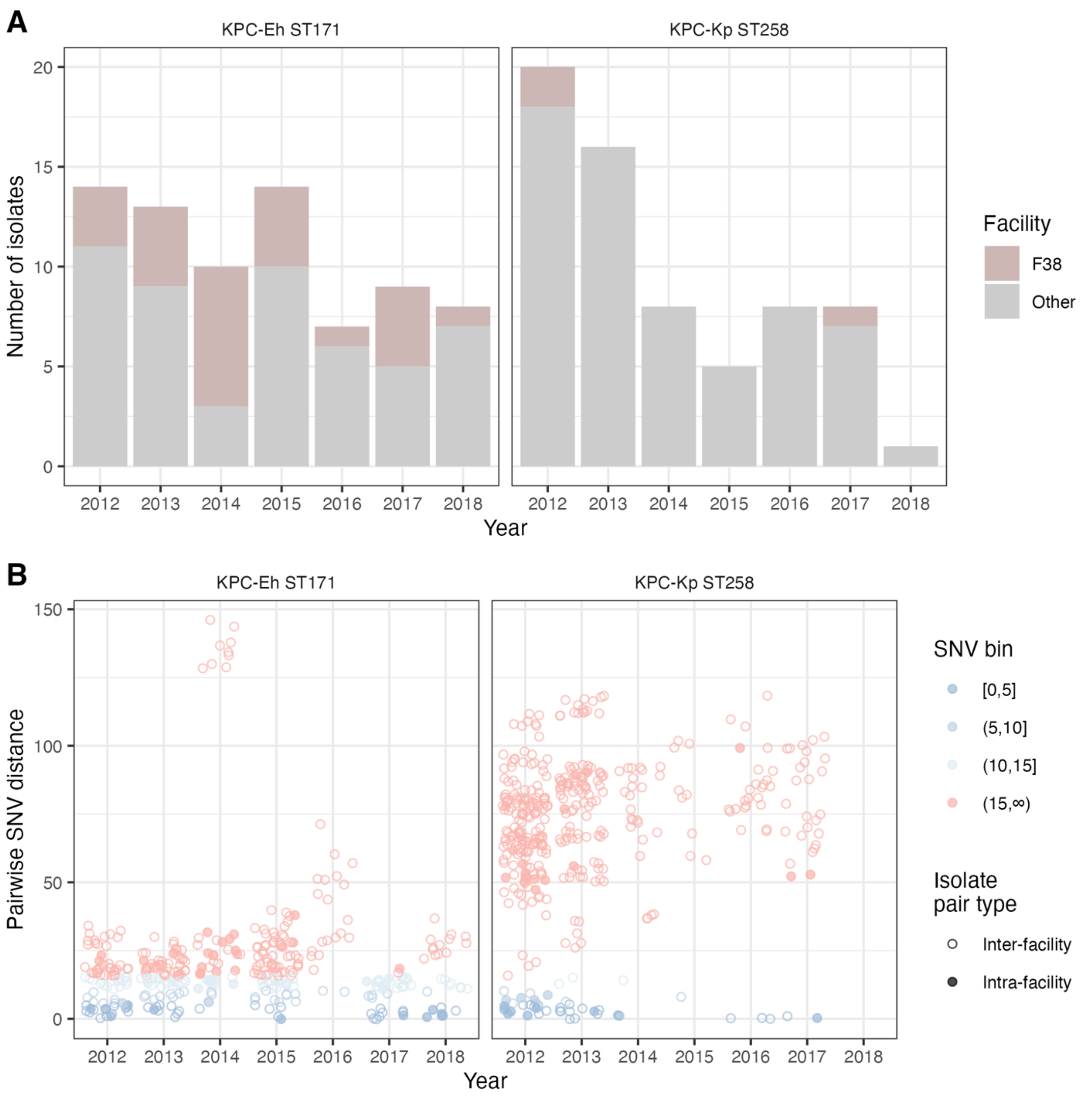

Figure S4: Minnesota (MN) isolates over time. (A) Number of ST171 and ST258 isolates over time. (B) Pairwise SNV distance of isolates over time. ST=sequence type; Eh=*E. hormaechei*; Kp=*K. pneumoniae*.

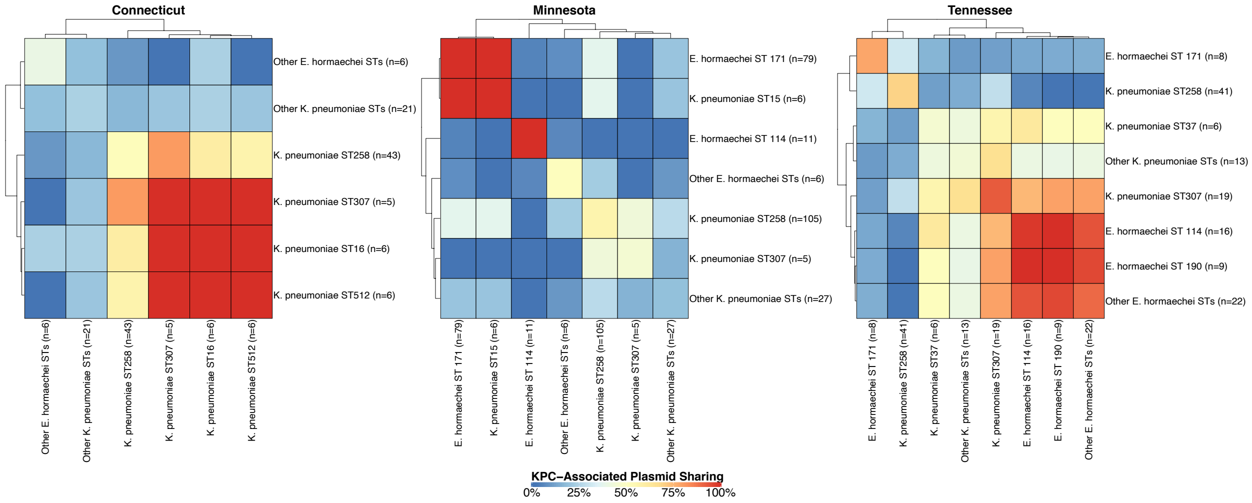

Figure S5: Pairwise plasmid cluster sharing. The proportion of plasmid sharing across two species/ST categories (heatmap fill) was calculated by dividing the number of isolate pairs across the two categories that shared a KPC-associated plasmid cluster by the total number of isolate pairs across the two categories. Note that KPC-associated plasmid clusters were considered to be shared irrespective of whether both isolates' KPC-containing contig were present on the plasmid cluster. Sequence types with less than 5 isolates were collapsed into "other" categories.
